# LK-1: an investigational therapy targeting hCG-β in metastatic breast, bladder, ovarian, and cervical cancers

**DOI:** 10.1038/s41598-026-38909-6

**Published:** 2026-02-20

**Authors:** J. Helena Kinion, Michael B. McAllister, James E. Summerton, Brian P. Dolan

**Affiliations:** 1 Morpholino Therapeutics, LLC, Philomath, OR USA; 2 Gary R. Carlson College of Veterinary Medicine, Oregon State University, Corvallis, OR USA

**Keywords:** Human chorionic gonadotrophin, hCG-β, RNA therapeutics, Antisense morpholino oligomers (PMO), Cancer research, Drug discovery, Cancer, Cell biology, Drug discovery

## Abstract

Human chorionic gonadotropin beta (hCG-β), while primarily recognized for its role in pregnancy, has also been implicated in poor prognosis for cancer patients over the past several decades. Targeting dysregulated hCG-β production by cancer cells may prove an effective way to combat tumor growth. We propose that targeting hCG-β production using antisense phosphorodiamidate morpholino oligomers (PMO) technology could provide a precise and effective strategy for lowering hCG-β levels. This method may offer enhanced specificity and the ability to modulate hCG-β expression directly at the mRNA transcript level. Here, we tested multiple PMO sequences targeted against hCG-β mRNA transcripts at various regions to ensure optimal sequence specificity and effective knockdown. We used cell viability assays, pregnancy test strips, and ELISA assays to determine knockdown by each PMO sequence to initially screen PMO sequences for identifying the most effective candidates. Of the sequences evaluated using ELISA, one demonstrated demonstrated effective knockdown at 0.5 µM and three at 1 µM. The remaining sequences required high PMO concentrations to observe a phenotype, and some did not produce measurable hCG knockdown under the conditions tested. Across nineteen PMO sequences tested in eight different cell lines, seven consistently reduced cell viability at 2-4 µM, whereas the remaining sequences showed variable or minimal effects. From the initial screen we identified the best PMO sequence, termed LK-1, for further analysis. We demonstrated that LK-1 is effective at significantly reducing hCG-β expression as measured both by western blot and ELISA assays. LK-1 treatment also showed significant reduction in cancer cell viability, reduce clonogenicity in colony formation assays, increased tumor cell death, and reduced invasion via wound healing assay in several cancer cell lines. We also showed that LK-1 is effective at reducing tumor formation in 3D spheroids. Combined, these findings suggest that targeting hCG-β production with morpholino-based therapy may represent a promising approach for cancers expressing this protein.

**Supplementary Information:**

The online version contains supplementary material available at 10.1038/s41598-026-38909-6.

## Introduction

Human chorionic gonadotropin (hCG) is a glycoprotein hormone that plays an important role during pregnancy^1^. hCG is a heterodimer that consists of an alpha and beta subunit and belongs to the glycoprotein family that consists of luteinizing hormone (LH), follicle-stimulating hormone (FSH), and thyroid-stimlulating hormone (TSH)^1^. Structurally hCG exhibits extensive charge heterogeneity due to different glycosylation states^1^. Cole^2,3^ defined the five variants of hCG: hCG, hyperglycosylated hCG (H-hCG), sulfated hCG (hCG-S), β subunit hCG (hCG-β), and H-hCG-β. hCG and H-hCG are virtually always expressed in placental and other trophoblastic tumors with occasional findings in nontrophoblastic tumors^1^. H-hCG-β/hCG-β have been found to be expressed in numerous nontrophoblastic epithelial cancers^1,4^ such as bladder^5–7^, breast^8^, pancreatic^9^, ovarian^10^, cervical^11–13^, and many more^5–9,13–35^. Its presence in tumors is often a hallmark of aggressive and metastatic disease that is associated with poor clinical outcomes^1,36–40^. Specifically, the expression of hCG-β has been associated with advanced tumor stage^38,40–42^, shorter disease-specific overall survival (OS)^38^, and resistances to chemotherapy and radiation treatment^33,39,43,44^. Several in vitro and in vivo studies have been conducted to investigate the involvement of hCG-β in cancer. These studies found that hCG-β plays a role in blocking apoptosis, promoting metastasis and invasion, evading the immune system, binding to transforming growth factor β (TGF-β) to induce angiogenesis, and binding to matrix metalloproteinases (MMPs) to promote invasion^6,12,45,46^.

hCG-β is the biologically active subunit that helps make up the heterodimeric form of hCG^2,47^. hCG-β is encoded by 6 CGB gene clusters on chromosome 19q13.32^47–49^ and its expression is regulated by several hormones (corticosteroids, progesterone, GnRH), growth factors (placental growth hormone, leukemia inhibitory factor, vascular endothelial growth factor (VEGF)), cytokines (Interleukin (IL)-6, epidermal growth factors (EGF), tumor necrosis factor (TNF)-α), ligands of the nuclear receptor PPARγ and the homeobox gene (DLX3)^50–53^. The beta subunit of hCG is encoded by CGB3, CGB5, CGB7, and CGB8 while CGB 1 and CGB2 are assumed to be pseudogenes^54^. While pseudogenes typically do not express functional proteins, it has been noted that CGB1 and CGB2 have four splice variants that could encode for small polypeptides^49,55^. These variants have been detected in certain epithelial cancers, suggesting they could potentially play a role in cancer development.

While the role of hCG-β in bladder, cervical, ovarian, choriocarcinomas, and other cancers have been well described for promoting carcinogenesis^56–58^, hCG-β function in breast cancer is controversial^59^. In vitro studies investigating hCG and hCG-β in human breast cancer have been reported to inhibit proliferation and induce differentiation suggesting a protective role in breast cancers^60^. In contrast, a recent study found that hCG had the opposite effect by stimulating proliferation in serum-free conditions^61^. Additionally, hCG was able to increase differentiation in cancer stem cells which could allow them to easily metastasize and invade other tissues^61^. Lastly, another study reported that breast cancer with BRCA1 mutations showed higher expression of hCG-β in vitro and in vivo and that BRCA1 transcriptionally regulated hCG-β expression^62^. It was also confirmed that hCG-β expression promotes tumorigenesis through TGF-βRII signaling^62^.

For over three decades, hCG-β has been investigated in the context of cancer therapy due to its role as a potential tumor marker and therapeutic target^63,64^. Clinical trials have explored the possibility of immunologically eliminating hCG-β by both vaccination using a C-terminal peptide (CTP) of hCG-β or adoptively transferring monoclonal antibodies to elicit an immune response against hCG-β expressing cancers^63,65^. However, translating these approaches into clinical use has proven challenging. hCG vaccines face specific difficulties due to the complexity of glycoproteins, their diverse subunit activities, and varying degrees of immunogenicity^66^. Additionally, the different forms, patterns, and degrees of glycosylation of hCG in vivo add further complexity^2,67–69^.

We have developed a therapeutic agent called LK-1, which specifically inhibits the translation of multiple variants of hCG and hCG-β. Unlike traditional treatments that use small molecules or antibodies to target specific regions of a protein, LK-1 blocks translation of the mRNA transcript of hCG-β using delivery enhanced phosphorodiamidate morpholino oligomers (PMO) technology and offers a promising way to disrupt hCG-β expression. Delivery enhanced PMOs like LK-1 may provide several advantages over traditional drug development methods, including protection from in vivo degradation, reduced risk of immune responses, high solubility, stability, and precise target specificity^70,71^.

Here, we tested various PMO sequences targeting different hCG-β transcripts, and from these experiments, we identified the optimal PMO sequence, LK-1, which effectively suppressed hCG-β expression. Further studies confirmed the specificity of LK-1 in targeting hCG-β, making it a promising anti-cancer therapeutic. LK-1 provides a targeted therapeutic approach for cancers that express hCG-β, including breast, bladder, ovarian, cervical, and other tumor types. We demonstrate that LK-1 can effectively knockdown hCG-β expression and that knockdown is correlated with reduced cell viability and increased apoptosis. LK-1 reduces migration, metastasis, invasion, and increases cell death in several cancer cell lines and impacts the formation of tumor spheroids. This study demonstrates that LK-1 may have the potential to be an effective approach in many types of cancers that express hCG-β.

## Results

### Optimal sequence targeting of hCG-β

Based on the genetic information from NCBI and Ensembl^72,73^, we designed suitable PMO sequences against hCG-β to aid in specific and efficient knockdown. While CGB3, CGB5, CGB7, and CGB8 have the same open reading frame, there are some differences in the 5′ UTR region which should be accounted for when trying to target more than one CGB gene using one PMO. CGB1 has two isoforms, CGB2 has two isoforms, and CGB7 has 3 isoforms. CGB1-201 and CGB2-201 have some sequence similarities, while CGB1-201 and CGB1-202 don’t have any sequence homology. CGB3, CGB5, CGB7-201, CGB7-202, CGB7-203, and CGB8 all have sequence similarities. Table 1 shows the different sequences tested against hCG-β. All designed PMO sequences are translation blockers and some PMOs only target one CGB gene. For example, PMO 1 and 2 target CGB1-201/CGB2-202 while PMO8-18 targets CGB3,5,7,8.

**Fig. 1:**
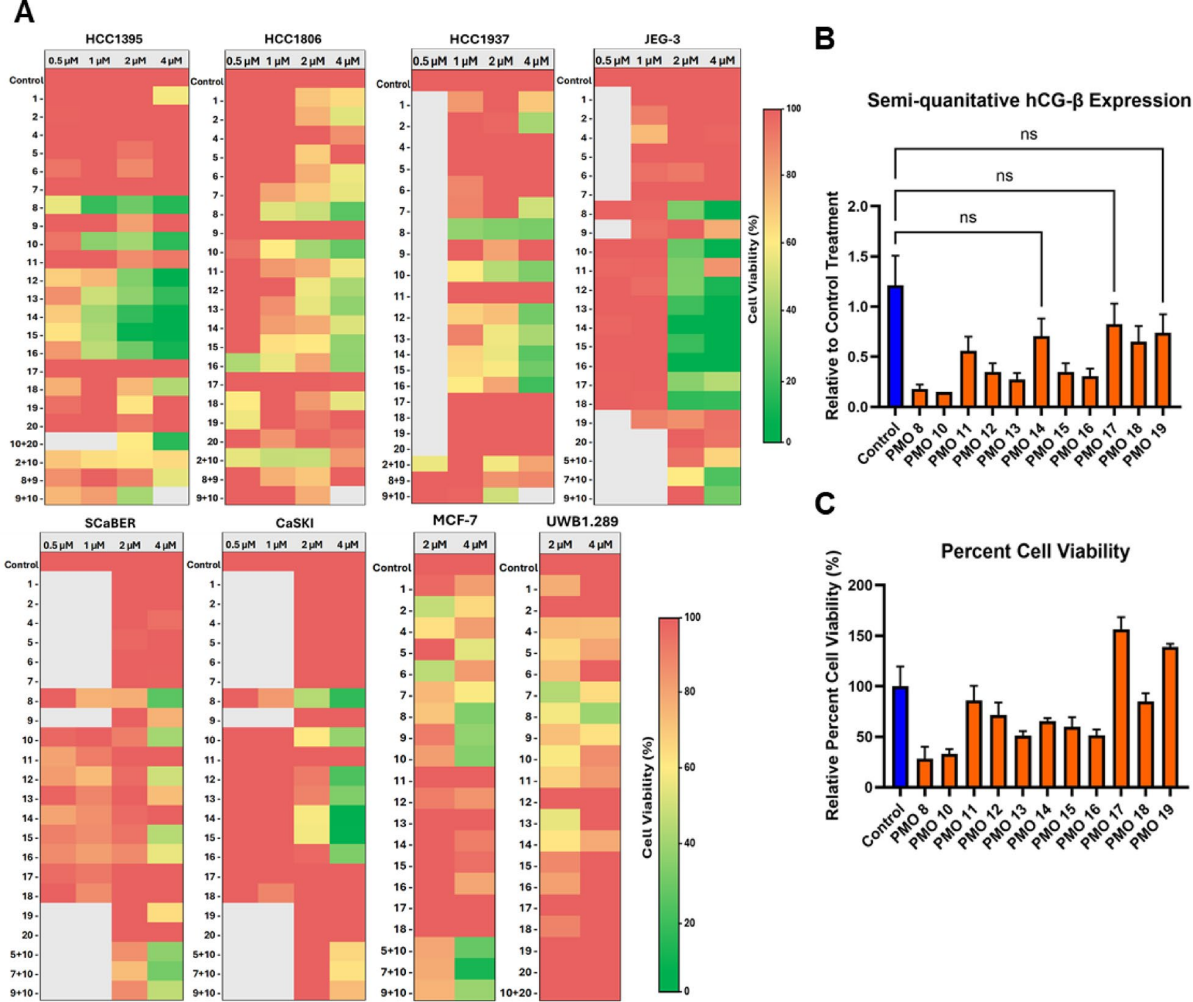
Identification of hCG-β targeting PMOs that reduce cell viability and decrease hCG-β release. **A** The TNBC HCC1395, HCC1806, HCC1937, CaSKI (Cervical), SCaBER (Bladder), MCF-7 (ER + BC), and UWB1.289 (Ovarian) cancer cell lines were tested against multiple individual PMO sequences and combinations at 0.5 µM, 1 µM, 2 µM, and 4 µM. Percent cell viability was calculated based upon cell titer glo viability assay after 72 h posttreatment along with a standard control morpholino sequence. Percent cell viability was made relative to the negative standard control vivo-PMO and a heat map was made to screen each morpholino sequence. The results are representative of four independent studies performed in quadruplicates. **B** JEG-3 cells were treated with various PMO sequences including a standard control morpholino as a negative control at 4 µM for 72 h prior to testing hCG-β expression using pregnancy test strips. Image J software was used to semi-quantitate the bands. Experiments are representative of three independent experiments performed in duplicates. **C** Treated the same as in A except, HCC1806 were used to investigate cell viability of several different PMO sequences treated using Cell Titer Glo and percent cell viability was made relative to the standard control. Experiments are representative of three independent experiments performed in triplicates.

**Fig. 2:**
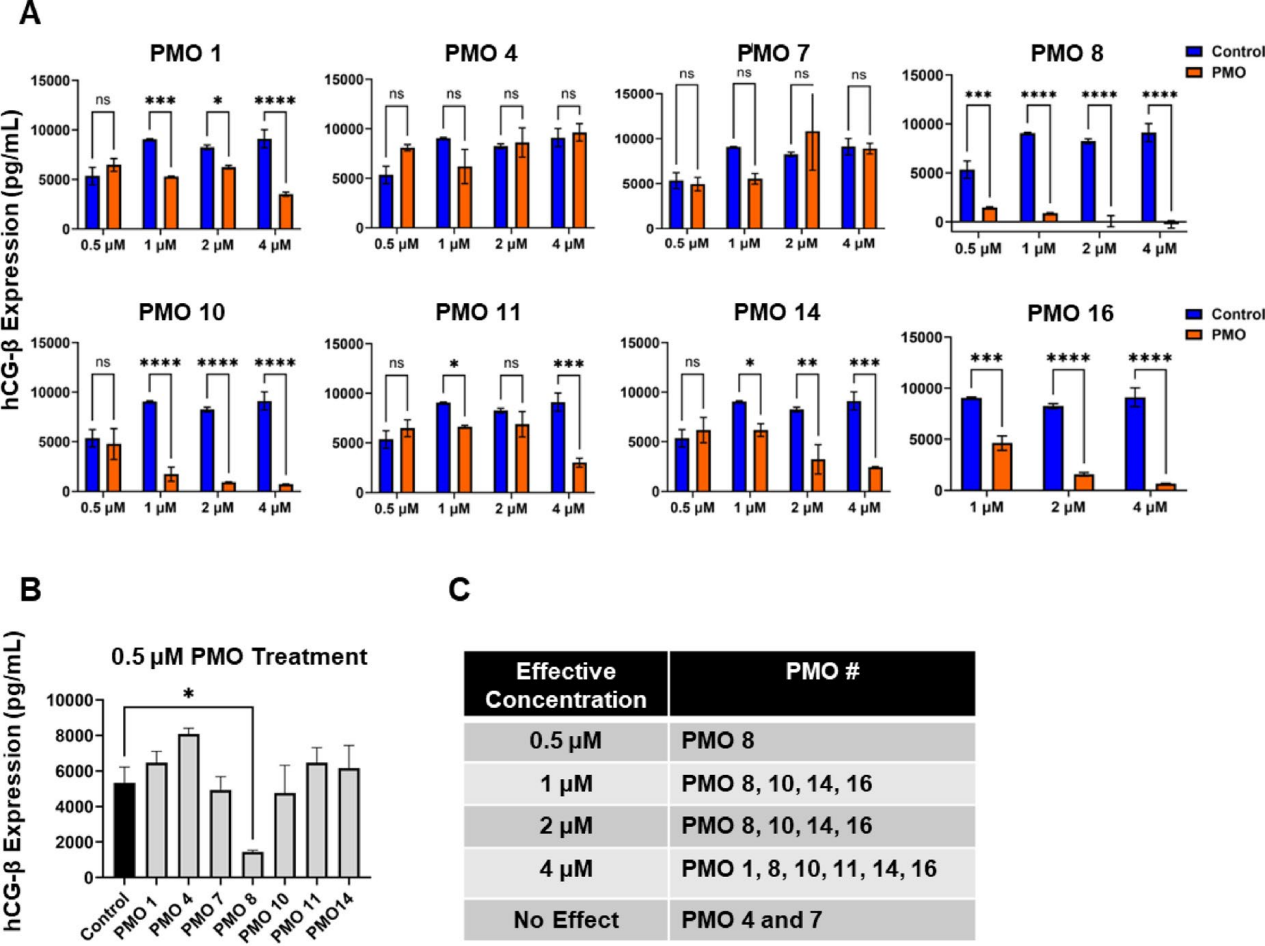
Variation in hCG-β knockdown by different PMOs targeting distinct regions of the hCG-β transcript at varying concentrations in SCaBER cells. **A** SCaBER cell lines were treated with a control PMO and 8 different PMOs that target various regions on the hCG-β mRNA transcript at their respective concentrations for 72 h. The media were then collected for ELISA to quantify ectopic hCG-β expression after treatment. Representative of two independent experiments performed in triplicates. Two-way ANOVA with Sidak multiple comparisons test, * *P* < 0.05, **P < 0.01, ***P < 0.001, ****P < 0.0001, ns, not significant. **B** Same as A, graph shows all the PMO sequences compared to control PMO at 0.5 µM. Representative of two independent experiments performed in triplicates. Ordinary one-way ANOVA with Dunnett multiple comparisons test, * *P* < 0.05. **C** Table showing the effectiveness of each PMO at different concentrations. PMO 8 (later renamed LK-1) was effective at all concentrations, while PMO 4 and PMO 7 showed no effectiveness at any concentration.

Next, we used Vivo-Morpholinos (Vivo-PMO) when looking at the sequence efficacy of each PMO to ensure cellular delivery. Vivo-PMOs uses an octa-guanidinium dendrimer (vivo) that is covalently bonded to the 3’ end of its targeted PMO to enhance cellular uptake^74,75^. We designed and tested 19 PMOs against hCG-β and compared each hCG-β targeted sequence to a standard control PMO with the same delivery moiety. Cell lines tested were cultured overnight in serum containing media and the following day, the serum-containing media was removed and replaced with serum-free media and cells were treated with the indicated PMO. This step is necessary to specifically examine the effect of endogenously synthesized growth factors, such as hCG-β without the confounding influence of growth factors in fetal bovine serum^61^. We determined not all PMO sequences were as effective using cell viability, pregnancy test strips, and ELISA assays (Figs. 1, 2). Previously, other antisense oligonucleotides (ASOs), such as short hairpin RNA (shRNA) and short interference RNA (siRNA), have been used to reduce hCG-β expression, leading to cell death^56,76^. Therefore, our initial screening of potential PMO sequences measured cell viability after PMO treatment. Several cancer cell lines, including SCaBER (bladder cancer), CaSKI (cervical cancer), and JEG-3 (choriocarcinoma) are known to be sensitive to hCG-β treatment and knockdown, making them suitable for this purpose. Additionally, triple-negative breast cancer (TNBC) cell lines, HCC1395, HCC1806, and HCC1937, which are negative for progesterone receptor (PR-), estrogen receptor (ER-), and HER2, the hormone-positive breast cancer cell line, MCF-7, and UWB1.289 (ovarian cancer) were also used to measure cell viability and hCG-β expression and knockdown. By measuring cell viability, we aimed to evaluate the efficiency of each PMO sequence and compared it to a standard control PMO, as some sequences may target a single transcript while others could affect multiple transcripts. From the 19 sequences tested, 7 vivo-PMOs consistently induced cell death at 2 µM and 4 µM in several cancer cell lines that express hCG-β after 72 h of treatment (Fig. 1A). Based on the heatmap, PMO 8, 10, 12, 13, 14, 15, and 16 showed the greatest reduction in cell viability across most of the cancer cell lines tested. Following the initial screening of 19 sequences, 11 PMOs were selected for further analysis of hCG-β knockdown based on their effects on cell viability (Fig. 1B). The expression of hCG-β was initially measured using pregnancy test strip and semiquantitative analysis was performed (Fig. 1B). Of all the PMOs tested, 7 PMO sequences showed greater than 70% reduction of hCG-β expression. Of those 7, 2 (PMOs 8 and PMO 10) had a greater than 85% reduction in hCG-β expression in JEG-3 cell line (Fig. 1B) and were the most lethal to HCC1806 cells (Fig. 1C). This data demonstrates that knocking down hCG-β affects the viability of cancer cell lines and highlights the value of designing multiple morpholino sequences to identify the most effective one for therapeutic use.

We further investigated several PMO sequences for their ability to knockdown hCG-β expression using ELISA assays compared to a standard control PMO (Fig. 2). SCaBER cells were used to quantitatively measure hCG-β expression of PMOs 1, 4, 7, 8, 10, 11, 14, and 16 at 0.5 µM, 1 µM, 2 µM, and 4 µM after 72 h of treatment (Fig. 2A). PMO 8 showed the most effective suppression of hCG-β at the lowest concentration, 0.5 µM (Fig. 2A and B), while PMOs 10, 14, and 16 didn’t show decrease hCG-β until 1 µM. PMO 11 didn’t show decrease hCG-β until 4 µM and PMO 4 and 7 showed no effect in hCG-β knockdown. The effective concentration data is summarized in Fig. 2C.

### Analysis of LK-1 as an anticancer therapy against multiple types of cancer

Based upon the above data, we selected PMO 8 as the optimal PMO sequence for further analysis and termed it LK-1. LK-1 consists of an antisense-oligo targeted against hCG-β  and a proprietary multi-component delivery system designed to deliver the PMO into the cytosol of the cells. For the remaining experiments, we examined the effectiveness of LK-1 compared to a standard control PMO with the same delivery moiety in several cancer cell lines. This included HCC1395, HCC1806, and HCC1937, MCF-7, UWB1.289, UWB1.289 + BRCA1, SCaBER, CaSKI, and JEG-3^56–58^. As a negative control, we selected T-24, known for its lack of hCG-β expression^7^.

**Fig. 3:**
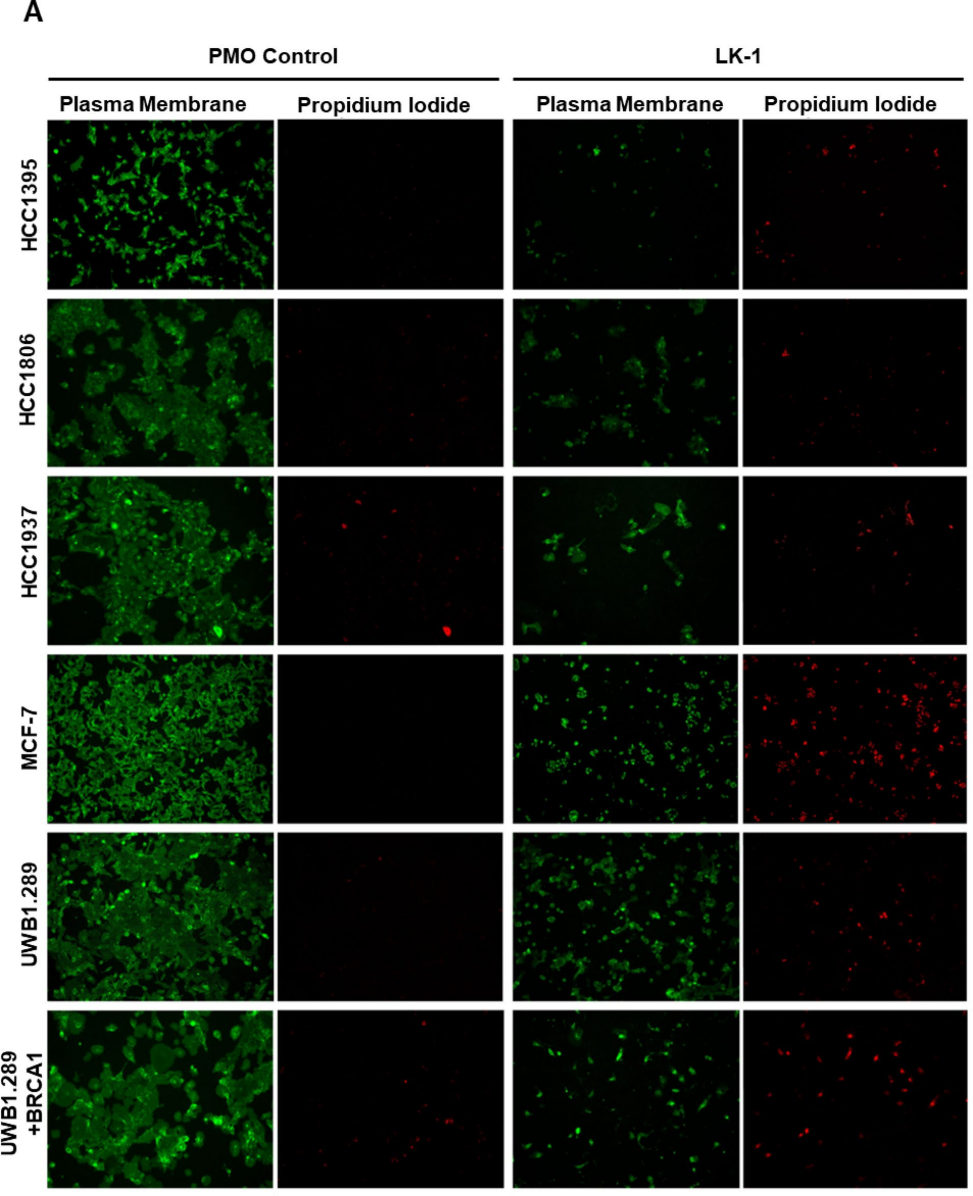

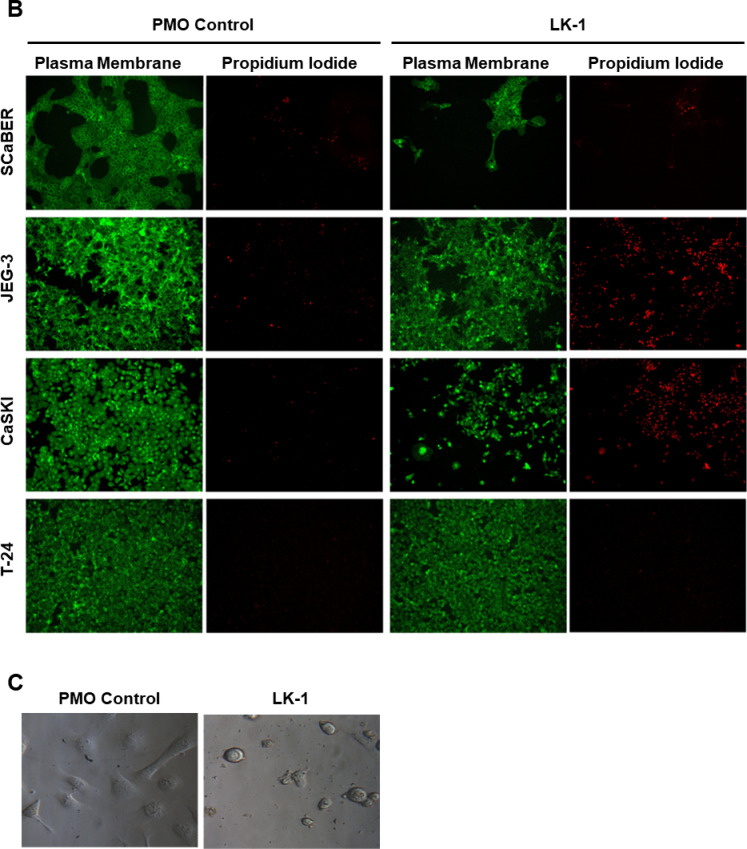
Fluorescent microscopy shows LK-1 decreases cell viability and increases cell death in hCG-β expressing cancers. **A** Live cell fluorescence microscopy data imaged at 10x magnification of HCC1806, HCC1937, MCF-7, UWB1.289, UWB1.289 + BRCA, SCaBER, JEG-3, CaSKI, and T-24 cancer cells following 72 h of treatment with control PMO or LK-1. Plasma membrane (green) was visualized with Leica DMIL LED microscope and imaged using Thorlabs monochrome camera to indicate the confluency of the cells and propidium iodide (red) to visualize dead cells. The images are respresentative of three independent experiments performed in triplicates. **B** CaSKI cells were treated with 4 µM of either a control PMO or LK-1 and imaged using a brightfield microscope at × 40.

We visually inspected tumor cell viability following treatment with either LK-1 or a negative standard control PMO. Each PMO was conjugated to the same delivery moiety and used at a concentration of 4 µM for 72 h. The cells were imaged after being stained with CellMask™ Plasma Membrane Stain (green) and propidium iodide (PI). As shown in Fig. 3A and B, all cancer cell lines, except for T-24, exhibited increased PI staining, a significant reduction in cell number, and morphological changes indicative of cellular distress. A high magnification light microscopy image of CaSKI cells (Fig. 3C) is shown as example of altered cellular morphology. Furthermore, these cell lines demonstrated significantly increased PI staining, suggesting higher levels of apoptosis or necrosis. While JEG-3 cells did not show morphological differences, those treated with LK-1 exhibited significantly higher propidium iodide (PI) staining compared to control-treated cells. HCC1806, HCC1937, UWB1.289, and SCaBER cells showed a marked reduction in cell count under LK-1 treatment compared to the control, along with an increase in PI staining, suggesting cellular damage resulting in cell death. In contrast, T-24 cells displayed no sensitivity to the treatment; both cell membrane integrity and PI staining were comparable to the control, indicating no adverse effects on this cell line.

**Fig. 4:**
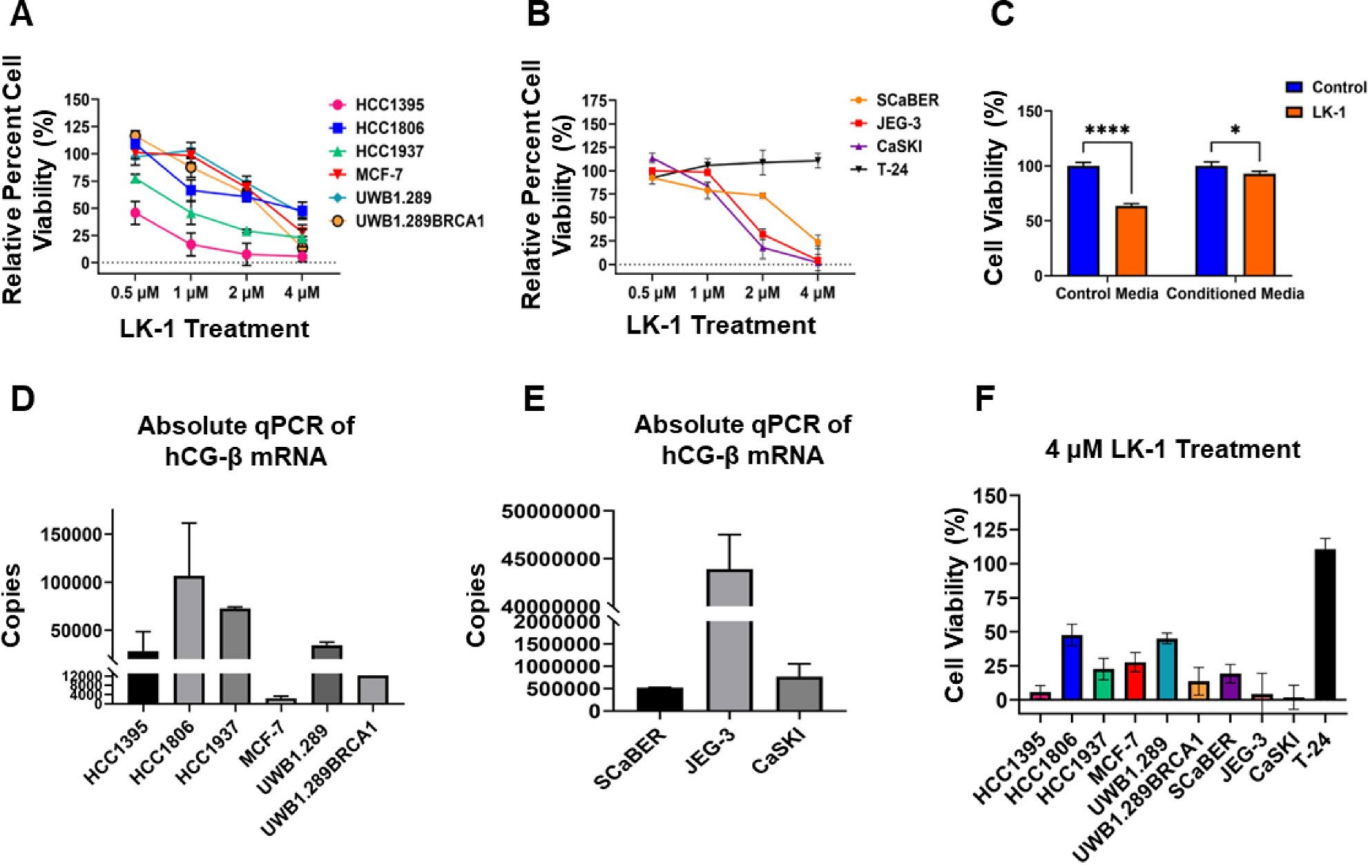
LK-1 reduces cell viability of cell lines that express hCG-β. **A** Relative percent cell viability determined by Cell Titer-Glo assay of HCC1395, HCC1806, HCC1937, MCF-7, UWB1.289, and UWB1.289 + BRCA1 cancer cell lines following 72 h treatment with LK-1 compared to control PMO. These experiments are representative of four independent experiments performed in quadruplicates. Two-way ANOVA with Sidak’s multiple comparisons test was performed in supplementary data to determine p values. **B** Same as A except using SCaBER, CaSKI, JEG-3, and T-24 cancer cell lines tested against LK-1 after 72 h of treatment show significantly reduced cell viability compared to the control PMO treatment. These experiments are representative of four independent experiments, performed in quadruplicates. Two-way ANOVA with Sidak’s multiple comparisons test was performed data to determine p values. **C** SCaBER cells were treated with either control PMO or LK-1 at 2 µM for 24 h, after which the treatment was removed and replaced with either conditioned media collected from untreated SCaBER cells or control media. The appropriate PMO was then added and cells were cultured for an additional 24 h. Cell viability was then assessed using Cell Titer-Glo. This experiment is representative of three independent experiments, performed in triplicates. Two-way ANOVA with Sidak’s multiple comparisons test was performed with * *P* < 0.05, ****p < 0.0001. **D** Absolute quantification of hCG-β and GAPDH as an internal control in HCC1395, HCC1806, HCC1937, MCF-7, UWB1.289, and UWB1.289 + BRCA1 was performed. RNA was collected and converted to cDNA and a standard curve was made using cDNA of known concentration following Azure Biosystems Cielo 3 RT-qPCR machine. Data is presented as mean +/- SD of triplicates of two independent experiments. **E** Same as E except using SCaBER, JEG-3, and CaSKI cell lines. **F** Summary of data from parts A and B, but shown as a bar graph to compare relative percent cell viability of all cell lines treated with LK-1 at 4 µM using Cell Titer Glo assay. This experiment is representative of four independent experiments performed in quadruplicates.

A dose-response assay was conducted using the Cell Titer-Glo assay to evaluate cell viability following a 72-h treatment of either the control PMO or LK-1 at varying concentrations (Fig. 4A, B). Our results demonstrate that LK-1 effectively induces cell death, leading to a dose-dependent reduction in cell viability across multiple cell lines except for T-24 which showed no response at any concentration. To demonstrate that cell-viability was related to loss of hCG-β, we used conditioned media from cells to rescue the lethality of LK-1 treatment. Conditioned media was isolated from SCaBER cells cultured in serum-free media for 24 h and the media was collected and filtered through a 0.22 µM filter. For rescue experiments, SCaBER cells were first plated overnight in media containing serum and the following day, treated with either LK-1 or standard control PMO in serum free media. After the first 24 h of treatment, the media was removed and replaced with either conditioned media containing the appropriate PMO or serum free media containing PMO. Cell viability was determined 24 h after the second treatment. As shown in Fig. 4C, the use of conditioned media, which contained hCG-β, restored cell viability.

We examined whether hCG-β levels correlated with lethality to LK-1.  Cell lines had a wide range of hCG-β mRNA levels (Fig. 4D, E), with JEG-3 expressing the highest levels and MCF7 the lowest. However, comparing levels of hCG-β transcript to LK-1 lethality (Fig. 4F) did not reveal any obvious pattern of mRNA expression to drug-induced lethality. For instance, HCC1395 were highly sensitive to LK-1, despite having lower hCG-β mRNA levels then HCC1806 and UWB1.289 cells. However, CaSKI and JEG-3, which had the highest level of hCG-β mRNA were the most sensitive to LK-1 treatment. These findings highlight LK-1’s potential to induce cell death across a broad spectrum of hCG-β expressing cancer types, including TNBC, ovarian, choriocarcinoma, bladder, and cervical cancers, irrespective of the relative amount of hCG-β mRNA.

**Fig. 5:**
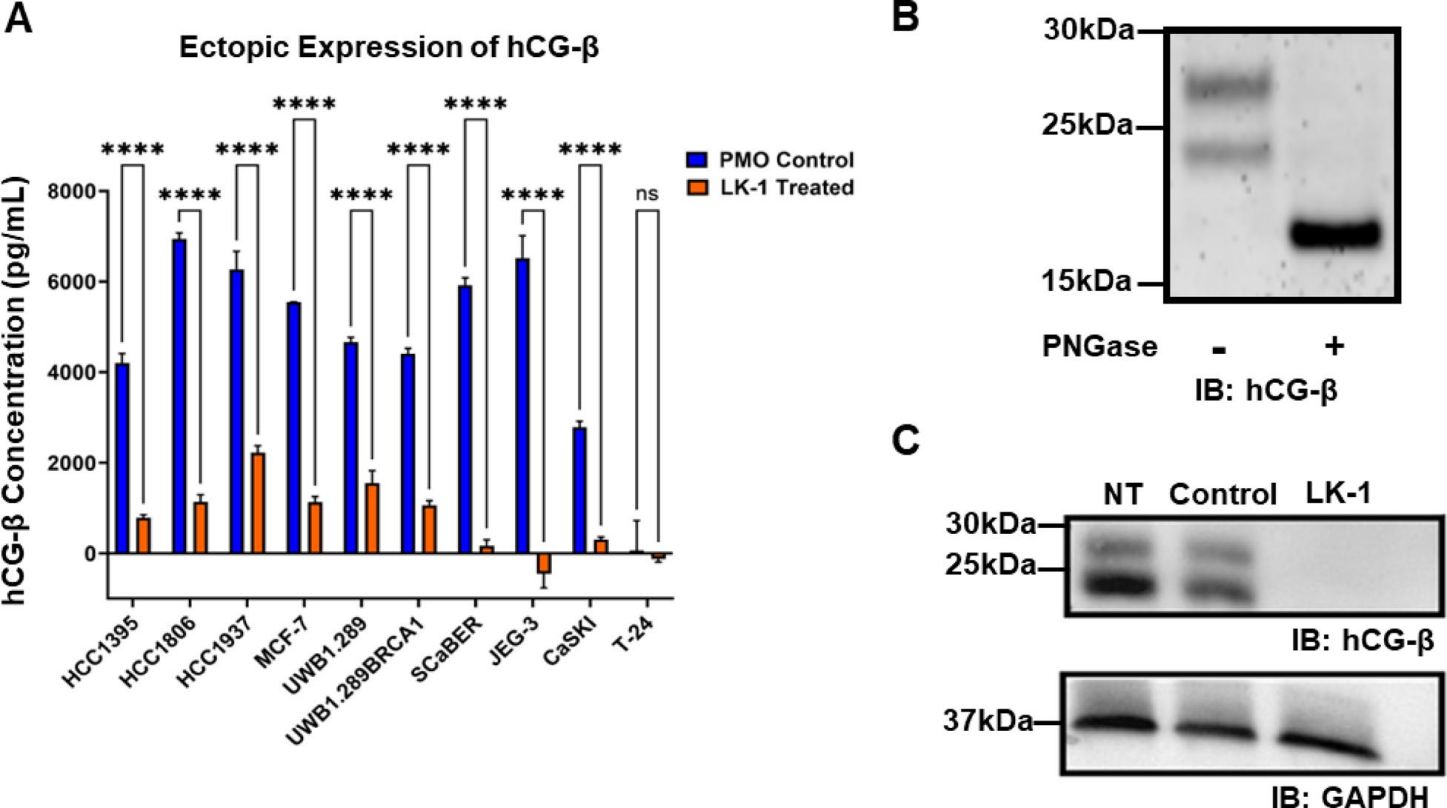
LK-1 reduces expression of hCG-β using ELISA and Western Blot assays. **A** HCC1395, HCC1806, HCC1937, MCF-7, UWB1.289, and UWB1.289 + BRCA1, SCaBER, CaSKI, JEG-3, and T-24 were treated for 72 h before culture supernatants were collected. Cell supernatant were analyzed in duplicates and mean +/- SD of three independent experiments are depicted. Two-way ANOVA with Sidak’s multiple comparisons test was performed with ns, not significant, ****p < 0.0001. **B** SCaBER cell lysates were either treated with PNGase F or left untreated, followed by Western blot analysis to assess hCG-β deglycosylation. **C** SCaBER cell line was treated with control PMO of LK-1 for 72 h and cells were collected, lysed, and analyzed by western blot analysis for either hCG-β or GAPDH. Western Blots were cropped to indicate specific bands.

Next we examined the impact of LK-1 treatment on the secretion of hCG-β in all tumor cell lines. Cells were treated with LK-1 or control PMO as above and hCG-β was measured by ELISA in the cell supernatant 72 h later. As shown in Fig. 5A, LK-1 greatly reduced secreted hCG-β from all tumor cell lines, except T-24 cells which do not express hCG-β. We then tested the impact of LK-1 on cell-associated hCG-β levels by western blot. Protein bands of approximately 28 kDa and 24 kDa were detected in SCaBER cells. Both bands were likely glycosylated as PNGase F treatment of cell lysates resulted in a single band approximately 22 kDa in size (Fig. 5B), demonstrating the effectiveness of detecting hCG-β in SCaBER cells. We then compared untreated SCaBER cells to cells treated with either the standard control PMO or LK-1. As shown in Fig. 5C, LK-1 treatment greatly reduced the levels of hCG-β detected in cells. These data further demonstrate that LK-1 can inhibit expression of hCG-β in tumor cell lines.

**Fig. 6:**
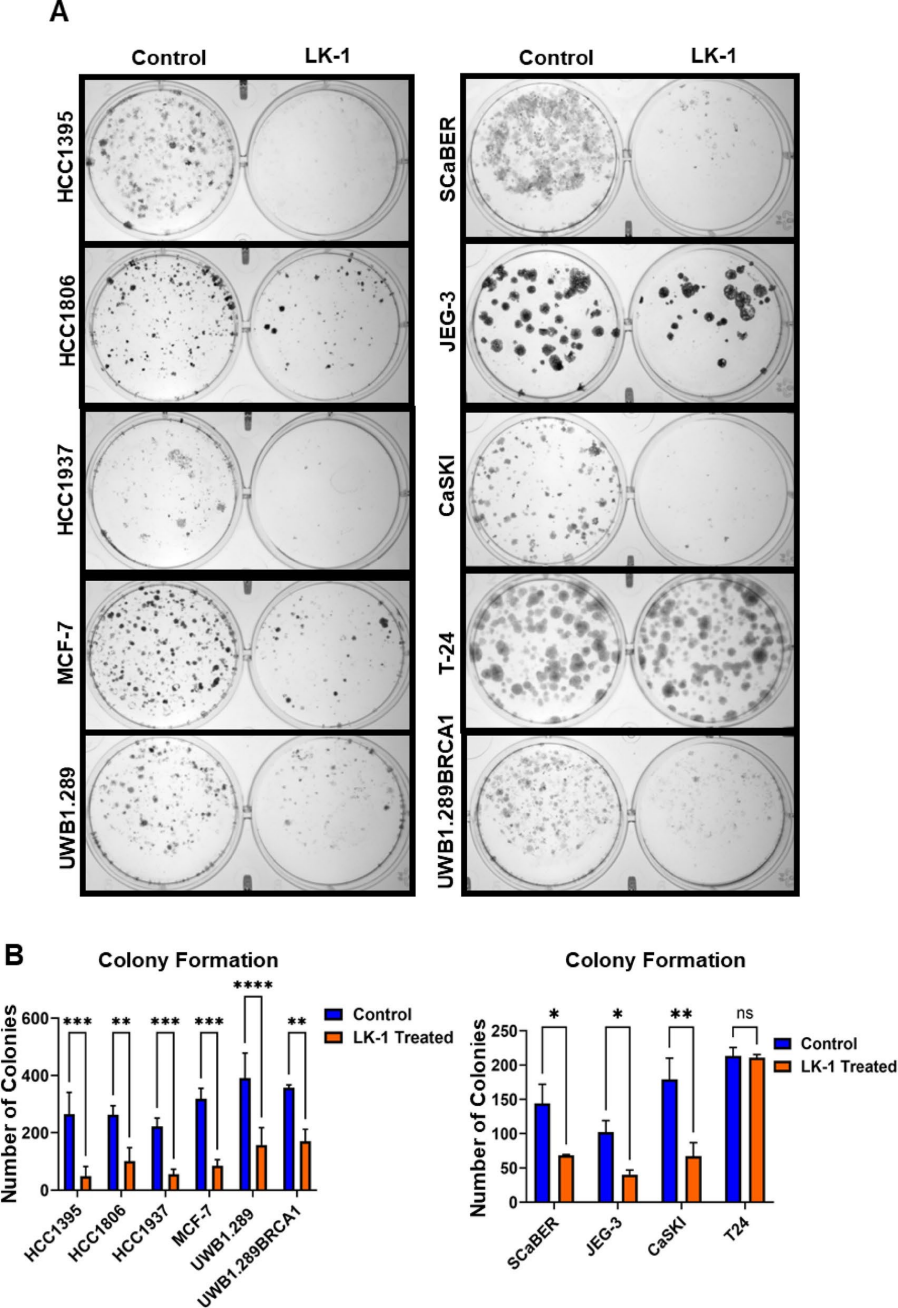
Effects of LK-1 on colony formation in cancer cell lines. **A** Representative images of colony formation of HCC1395, HCC1806, HCC1937, MCF-7, UWB1.289, UWB1.289 + BRCA, SCaBER, JEG-3, CaSKI, and T-24 cells lines following treatment of control PMO or LK-1. Cells were treated for one week, then cultured without drug for an additional week prior to visualization. and cell colony count data are presented as mean +/- SD of four independent experiments, each performed in duplicates. **B** Quantification of colony formation using imageJ in all of the cell lines listed in A. Cell colony count data are presented as mean +/- SD of four independent experiments, each performed in duplicates. Two-way ANOVA with Sidak’s multiple comparisons test was performed with ns, not significant, *p < 0.05, **p < 0.01, ***p < 0.001, ns, not significant.

We then investigated the capacity of cancer cells to form colonies following treatment with LK-1 compared to the control PMO (Fig. 6). All cell lines were treated with LK-1 for one week, after which the media was replaced, and the cells were cultured for an additional week in the absence of LK-1 before imaging. The results demonstrated that LK-1 significantly reduced clonogenic potential in several cancer cell lines, specifically, HCC1395, HCC1937, SCaBER, and CaSKI had the most sensitivity to treatment (Fig. 6A, B). Interestingly, UWB1.289 and UWB1.289 + BRCA1 had similar reductions in colony count (Fig. 6A) and suggest no discernible differences despite UWB1.289 having a higher expression of hCG-β compared to UWB1.289 + BRCA1. T-24 cells showed no colony count differences between the control treated and LK-1 treatment (Fig. 6B).

**Fig. 7:**
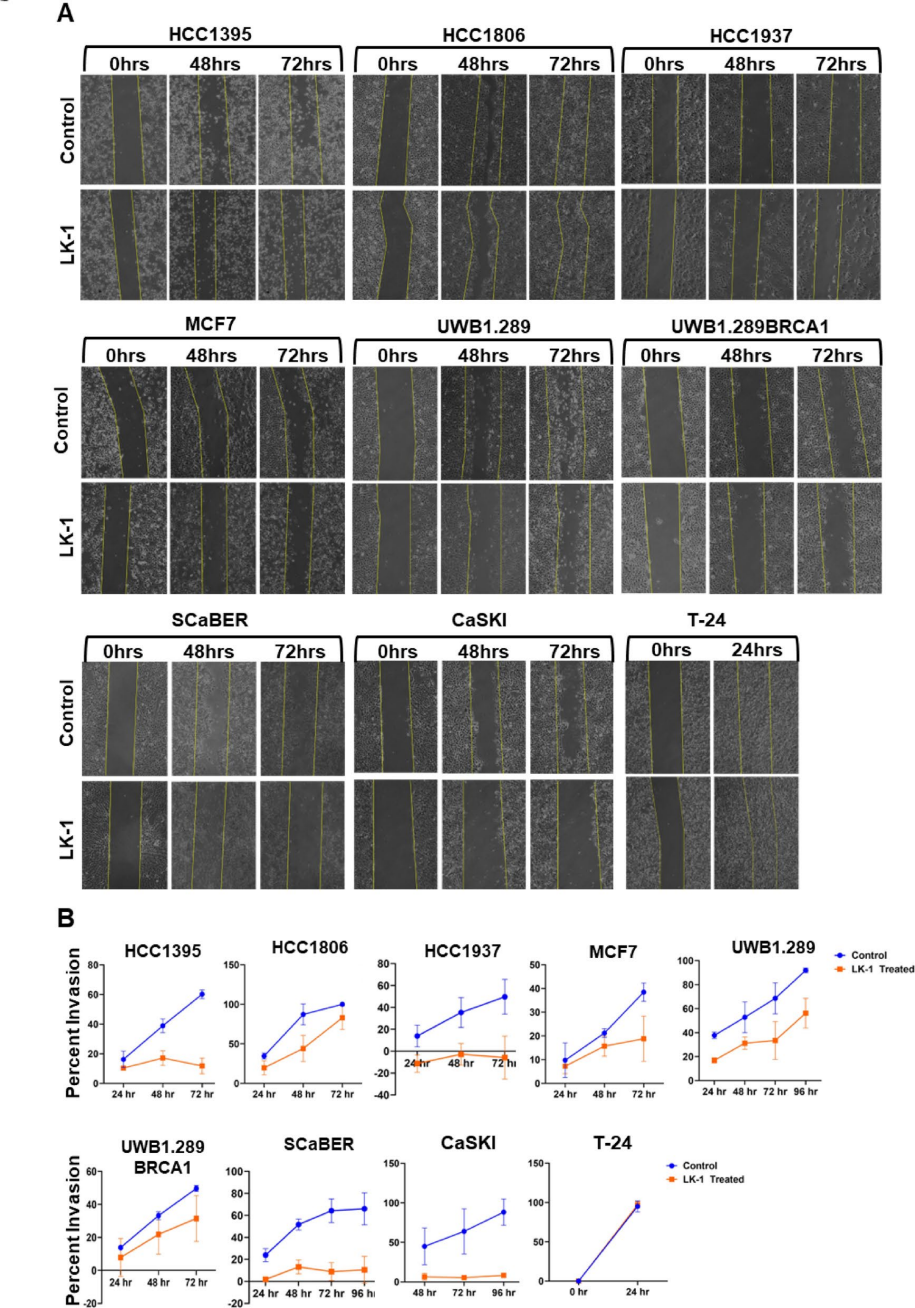
Effects of LK-1 on cell migration and invasion via scratch invasion assay. **A** Representative images of scratch invasion assay of HCC1395, HCC1806, HCC1937, MCF-7, UWB1.289, and UWB1.289 + BRCA, SCaBER, CaSKI, and T-24 cell lines. A sterile 200 µL pipette was used to scratch the cell to form a wound. Cells were then treated with LK-1 or control PMO for 24 h before the media was removed and washed with PBS and imaged on the indicated times. **B** Quanitiative measurements of cells growing over the line were quantified using ImageJ software. All tests were performed in triplicates and presented as mean +/- SD.

We aimed to investigate the effect of LK-1 on the migration of cancer cells using a scratch invasion assay (Fig. 7). Importantly, the cancer cells were treated with LK-1 or control PMO for only 24 h during the initial phase of the scratch assay, after which the treatment was discontinued for the remainder of the experiment. Our results indicate that LK-1 treatment inhibited cell migration in several breast cancer cell lines. However, the HCC1806 cell line exhibited a less pronounced reduction in migration at the 72-hour mark (Fig. 7A, B). We observed a significant decrease in migration in CaSKI and SCaBER cell lines (Fig. 7A, B), suggesting a stronger response to LK-1 treatment in these lines relative to certain breast cancer cell lines. Conversely, the T-24 cell line did not show any response to LK-1 treatment and had full migration by 24 h (Fig. 7). Overall, these findings demonstrate that LK-1 effectively reduces cell migration in various cancer cell lines, indicating its potential as a therapeutic agent in the management of cancer metastasis.

**Fig. 8:**
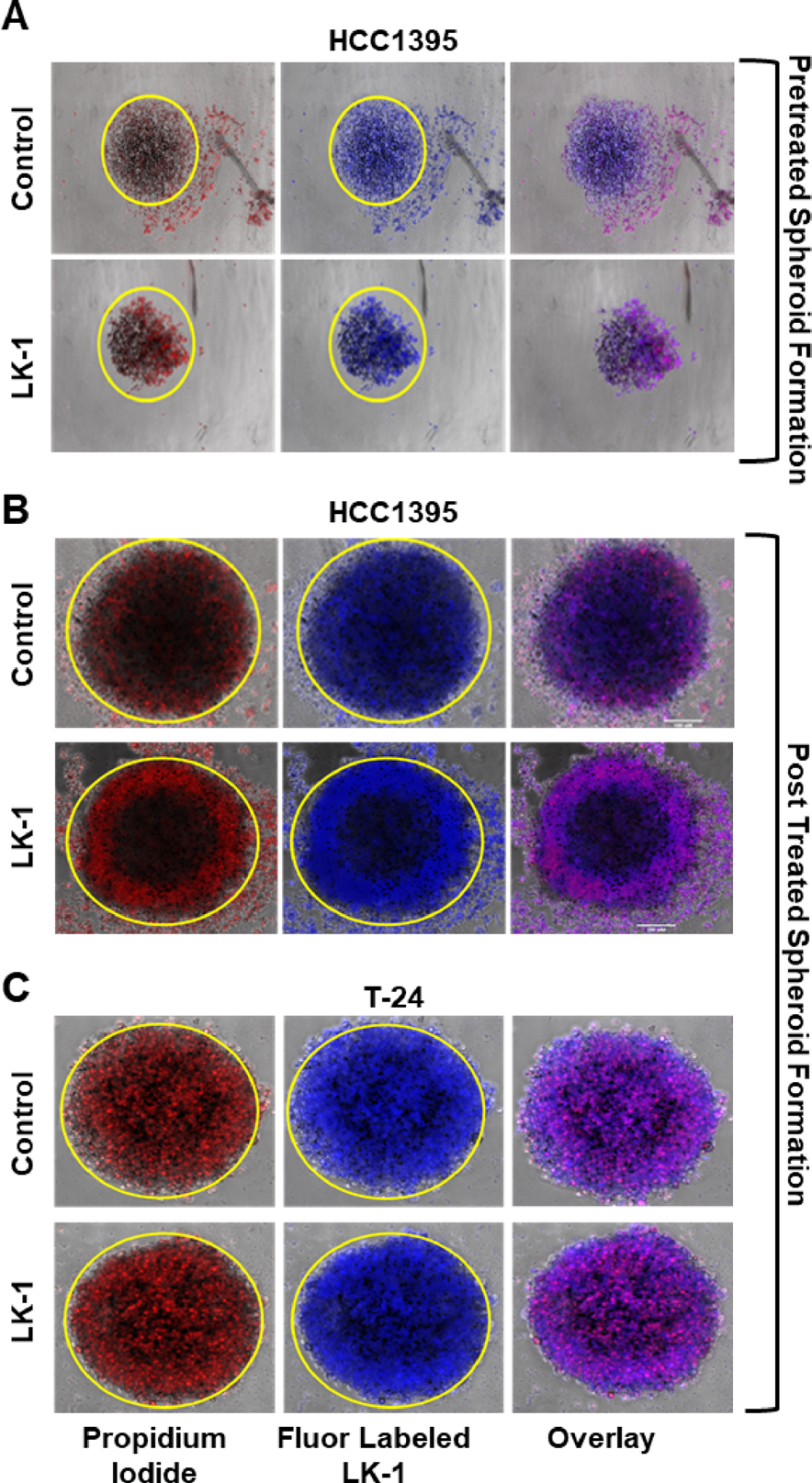
LK-1 impacts the formation of 3D spheroid formation and shows hCG-β act as an autocrine in 3D tumor spheroids. **A** HCC1395 cells were treated for 2 h at 2 µM with fluorescently labeled LK-1 or control PMO prior to formation of 3D tumor spheroids. PMOs were visualized by the addition of FITC to the PMO (false-colored blue). Spheroids were also stained with propidium iodide (red) to track cell death. Images are from 96 h post formation and show either a reduction in tumor size or more cell death with irregular shape in HCC1395 cell line. Tests are representative of four independent experiments. **B** HCC1395 spheroids were allowed to form for 24 h before continuous treatment with 4 µM fluorescently labeled LK-1 or control PMO. Spheroids were also stained with propidium iodide (red) to monitor cell death. Representative images taken at 144 h post-treatment. Tests are representative of four independent experiments. **C** Similar to B except T-24 cells were used in place of HCC1395. Tests are representative of four independent experiments each performed in duplicates.

We tested the ability of LK-1 treatment to impact 3-dimensional tumor spheroids in vitro in pre- and post-treatment assays (Fig. 8). HCC1395 cells were treated with fluorescently labeled (blue) LK-1 or control PMO for 2 h prior to a 96-h incubation to allow spheroids to form. PI was also added 24 h after formation to visualize cell-death. As shown in Fig. 8A, pre-treatment of HCC1395 cells with LK-1, but not the control PMO, partially inhibited spheroid formation, and increased staining of PI in the spheroid. However, investigation of the treatment of LK-1 after tumor spheroids formation in HCC1395 didn’t show much size or shape difference after 144 h post treatment (Fig. 8B). This could suggest the drugs lack of ability to effectively penetrate the spheroid. The spheroids were stained with PI and LK-1 and the control PMO were fluorescently tagged (blue) in order to track the drug in the spheroid models. Additionally, there was a notable increased staining for PI, suggesting that LK-1 treatment may induce tumor cell death on the periphery of the tumor spheroid. Interestingly, it was observed that there was a ring on the outer layer of the spheroid in the LK-1 treatment in HCC1395 while there was no ring in the T-24 cell line (Fig. 8B, C). This could suggest that hCG-β is secreted in the cells around the outer surface which show hCG-β acting as an autocrine (Fig. 8B). However, further investigation would be needed to determine that. Additionally, while there was no reduced spheroid size after treatment of LK-1, there was an increase staining for PI, suggesting that LK-1 treatment is inducing more cell death compared to the control treated spheroid.

**Fig. 9:**
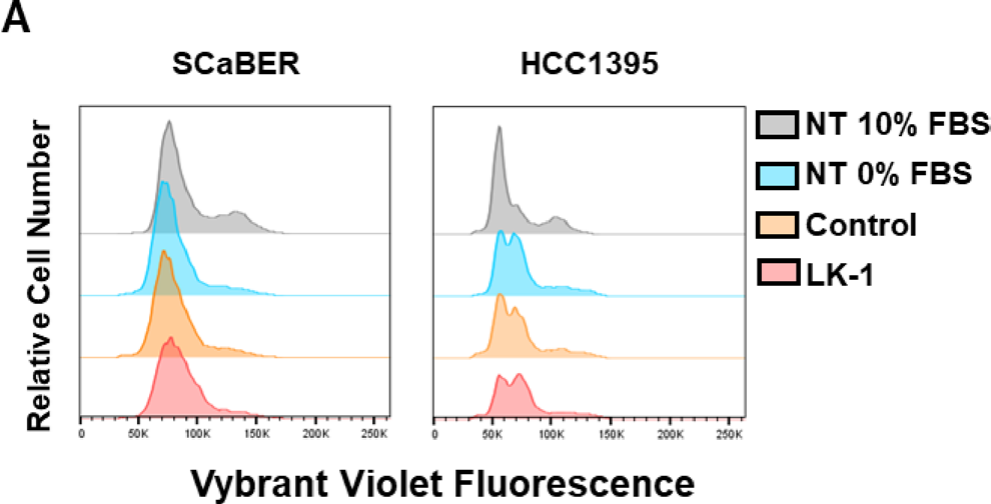
Cell cycle analysis of SCaBER or HCC1395 cells during LK-1 treatment. SCaBER and HCC1395 cells were treated with 4 µM LK-1 or control PMO. After 24 h (HCC1395) or 48 h (SCaBER), cells were stained with Vybrant DyeCycle Violet at 37 °C for 30 min and analyzed by flow cytometry. Data shown are representative of three independent experiments, each performed in triplicate.

Finally, we examined the effect of LK-1 treatment on cell cycle progression by measuring DNA content with a fluorescent, cell-permeable dye, Vybrant DyeCycle Violet and analyzing cells by flow cytometry. As shown in Fig. 9, both HCC1395 and SCaBER cells demonstrated fairly standard G1/G0-S-G2 distribution with approximately 15–20% of cells in G2 phase. Following overnight treatment in serum free media, the cell cycle profile shifts for both cell types, as would be expected by removing the serum-derived growth factors. Further treatment with LK-1 or standard control morpholinos did not further alter the cell cycle in either cell type. These data indicate that loss of hCG-β did not appear to alter cell cycle progression.

## Discussion

Here we describe a method to inhibit translation of hCG-β expressed by tumor cells to inhibit tumor cell growth. PMOs, a class of synthetic antisense oligomers, have emerged as a promising tool in drug development due to their ability to selectively modulate gene expression^71,77^. Their unique chemical structure provides stability against nucleases and facilitates the targeting of specific mRNA sequences, making them valuable for therapeutic applications, particularly in the context of genetic diseases and cancer^70^. In this study, we investigated several morpholino targets for efficacy, knockdown, and viability in breast, bladder, choriocarcinoma, and ovarian cancers. We identified several PMOs that reduced cell viability and expression of hCG-β. The most efficacious PMO, named LK-1, was used for further study. The ability of LK-1 to effectively knock down hCG-β expression correlates with significant reductions in cell viability and increased cell death, suggesting that hCG-β plays a crucial role in tumor cell survival and proliferation. This aligns with previous research indicating that elevated hCG-β levels are associated with aggressive tumor behavior and poor prognosis in cancer patients^76,78^.

Our results demonstrate that LK-1 not only reduces cell viability but also inhibits critical cancer hallmarks such as migration, invasion, and metastasis. The observed decrease in cell migration and invasion in various cancer cell lines, particularly in triple-negative breast cancer (TNBC) and cervical cancer cell lines, underscores the potential of LK-1 as a multi-faceted therapeutic agent. These findings are consistent with literature suggesting that hCG-β contributes to the epithelial-mesenchymal transition (EMT) and metastatic potential of cancer cells^76,79^. The ability of LK-1 to diminish these processes could provide a strategic advantage in managing cancers characterized by hCG-β expression.

Interestingly, our study also revealed that LK-1’s effectiveness is not solely dependent on cancers that exhibit higher levels of hCG-β expression. While TNBC cell lines exhibited higher hCG-β mRNA levels compared to ER + BC MCF-7 cells, LK-1 was effective across all of the hCG-β expressing cell lines tested, including those with lower expression levels. It should be noted that sensitivity to LK-1 did not necessarily correlate with expression of hCG-β. HCC1806 and UWB1.289, which both express high levels of hCG-β, seemed to be less sensitive to treatment in all assays measured. This could suggest that treatment may need to be more frequent or for longer periods of time compared to cancers that are expressed at lower levels. Additionally, this could also mean that these cell lines rely on other pathways unrelated to hCG-β for cell growth and survival. Taken together, these findings suggests that LK-1 may have a broader applicability than previously anticipated, potentially targeting various cancer types that express hCG-β, regardless of the expression intensity^78^. It should be noted, that tumors that do not express hCG-β are unlikely to be treated with LK-1 as the hCG-β negative cell line T-24 was unaffected by LK-1 treatment in all assays performed. 

The impact of LK-1 on tumor spheroid formation and size indicates its potential to disrupt proper formation of a tumor. In addition, the observation of a fluorescent ring around the treated spheroids may suggest that hCG-β functions in an autocrine/paracrine manner in a 3D spheroid model, promoting tumor growth and survival as previously described^80^. This finding warrants further investigation into the mechanisms by which hCG-β influences tumor microenvironment dynamics and how LK-1 can be optimized for deeper tissue penetration.

Worth noting, this study is limited by its exclusive use of in vitro models. While these systems provided valuable mechanistic insights, they do not fully recapitulate the complexity of the tumor microenvironment, including immune cell interactions, stromal contributions, vascularization, and drug metabolism. Consequently, the therapeutic potential of LK-1 remains to be validated in vivo, where factors such as bioavailability, tissue-specific distribution, and pharmacokinetics may significantly influence its efficacy. Future studies employing appropriate in vivo models, including murine xenografts or orthotopic tumors, will be essential to confirm and extend these findings.

The direct targeting of hCG-β mRNA may represent a streamlined and potentially promising therapeutic avenue compared to immunization strategies or adoptively transferred monoclonal antibodies. Our findings provide preliminary evidence that LK-1, an investigational antisense therapeutic, has potential for targeting hCG-β in multiple cancer types. By employing this antisense approach, LK-1 may help overcome some limitations of traditional antibody-based therapies by directly downregulating hCG-β expression. As we continue to refine this therapeutic modality, further research such as in vivo studies are essential to explore the full spectrum of LK-1’s effects and its potential integration into existing cancer treatment paradigms.

## Materials and methods

### Cell lines

Cell lines (HCC1395, HCC1806, HCC1937, MCF-7, UWB1.289, UWB1.289BRCA1, SCaBER, JEG-3, T-24 and CaSKI) were purchased from American Type Culture Collection (ATCC, Manassas, VA, USA) and maintained according to manufacturer’s instructions. HCC1395, HCC1937, UWB1.289BRCA1, and CaSKI were cultured in RPMI media (ATCC, Manassas, VA USA) containing 10% FBS (Summerlin Scientific, Hampton, New Hampshire, USA), 100 U/mL penicillin, and 100 mg/mL streptomycin (ATCC). UWB1.289, MCF-7, JEG-3, and SCaBER were cultured in EMEM media with 10% FBS (Summerlin Scientific, ), 100 U/mL penicillin, and 100 mg/mL streptomycin (ATCC). UWB1.289 and HCC1806 were cultured in DMEM media containing 10% FBS (Summerlin Scientific, ), 100 U/mL penicillin, and 100 mg/mL streptomycin (ATCC). T-24 were cultured in McCoy’s media containing 10% FBS (Summerlin Scientific), 100 U/mL penicillin, and 100 mg/mL streptomycin (ATCC). All cell lines were maintained at 5% CO_2_ at 37℃.

### LK-1 treatment conditions

PMOs, vivo-PMOs, and PMOs with a 5′ fluorescent tag were purchased from Gene Tools, LLC (Philomath, OR). Special modifications to add the proprietary delivery moiety were done and quality controlled before running experiments. For the control PMO, + 11 standard control (GCAATATGAAACCTCTTACCTCAGT) and LK-1 PMO sequence (GCAGCAGCCCCTGGAACATCTCCATC) was used with the same delivery moiety and both PMO’s were dissolved in water at 0.5-1 mM stock concentrations. Typical final concentrations that were used in each experiment were 2 µM and 4 µM.

**Table 1:** Sequences and properties of each PMO tested.

PMO	Sequence
1—CGB1-201, CGB2-202	GCAACAGCAGCAGCCTCTTTGACAT
2—CGB1-202, CGB2-202	CCATGCTCAGCAGCAGCAACAGCAG
4—CGB1-201 SB	GGATCTACCCTACCTTTGACATG
5—all CGB7 sb	GCCCTGCAGTCTTACCTGGAACATC
6—CGB2-201 AUG	GCAACAGCAGCAGCCCCTTTGACAT
7—CGB2-201 5′ UTR	CATGTCTCTCTCTTAGCGGGAT
8—B3-1, B5-1, B7-#3, B8-1	GCAGCAGCCCCTGGAACATCTCCATC
9—B5-1, B7-#3, B8-1 ATG23	CCCTGGAACATCTCCATCCTTGG
10—many hCGb 3,5,7,8	CAGCAGCCCCTGGAACATCTCCATC
11—hCG-β (3–8)	CAGCAGCCCCTGGAACATCTCCATCC
12—hCG-β (3–8)	GCAGCCCCTGGAACATCTCCATCCT
13—hCG-β (3–8)	CAGCAGCAGCCCCTGGAACATCTCC
14—hCG-β (3–8)	GCAACAGCAGCAGCCCCTGGAACAT
15—hCG-β (3–8)	CAGCAGCAGCCCCTGGAACATCTCCATC
16—hCG-β (3–8)	CAACAGCAGCAGCCCCTGGAACATCTCC
17—hCG-β (3–8)	CAGCAGCCCCTGGAACATCTCCATCCTTGG
18—hCG-β (3–8)	AACAGCAGCAGCCCCTGGAACATCTCCATC
19—CGB1-2 (201)	CATGCTCAGCAGCAGAACAGCAGC
20—CGB1-2 (202)	AGCGGGATATCTTCCGCAAGCACTGG

### Cell viability analysis

Cells were plated at 50k cells per well in 24-well plates and were allowed to adhere overnight. Cells were then treated in 0% FBS (serum free) media and incubated for 72 h prior to analysis. In some experiments, conditioned media obtained from cells cultured overnight in serum-free media and filtered through a 0.22 µM filter was used in conjunction with PMO treatment. Cells were washed, trypsinized, and transferred to a 96-well white TC-treated culture plates prior to running the assay. Additionally, 1 mL of media was added to each 24-well and 100 µL was added to each 96-well in quadruplicates. At the end of each treatment, CellTiter-Glo^®^ Luminescent Cell Viability Assay (G7570, Promega, Madison, WI, USA) was added to each well according to the manufacturer’s instructions. After the 5-minute incubation time, the luminescence was measured using BMG Labtech FLUOstar OPTIMA microplate reader (Cary, North Carolina, USA). The samples were then analyzed, and the percentage of viable cells were made relative to the control treated cells.

### Pregnancy tests

Ectopic expression of hCG-β in the media was initially determined using First Response™ pregnancy test strips (Walmart or Amazon) in order to measure hCG-β expression and knockdown in various cancer cell lines. The First Response™ brand was chosen because it was the only pregnancy test strip that confirms detection specifically against hCG-β along with other variants of hCG. Because the detection limit of these pregnancy tests, only cancer cell lines that generate a high abundance of hCG-β will result in a positive test and other analysis was performed such as ELISA and rt-qPCR. The test strips were performed according to manufacturers instruction and imaged. The images were then analyzed using ImageJ software for semi quantitative measurement and normalized to the control treatment.

### Western blotting

Standard western blotting was performed for analysis of protein abundance. Cell lysates were collected using RIPA lysate buffer with 10% Pierce™ protease inhibitors (Thermofisher, USA, A32955) and 2% phosphatase inhibitor (0.1 M Na_3_VO_4_ in DMSO). Pierce™ BCA Protein Assay Kit (Thermofisher, USA, 23227) was then ran to quantify the amount of protein collected. Once the amount of protein was determined, protein samples were deglycosylated using PNGase F (New England Biolabs, Beverly, MA) according to manufacturer’s instructions. Final prepared lysates were mixed in a 1:1 mixture of 2x Laemmli buffer with β-mercaptoethanol (BioRad) and boiled for 5 min prior to running on a 12% SDS PAGE gel (BioRad). After that, the gel was transferred to a PVDF membrane using BioRad Trans-Blot Turbo System. Blots were probed with the following antibodies: Primary antibody hCG-β Rabbit PolyAb at 1:500 (ProteinTech, 11615-1-AP, Thermofisher) and Secondary: 1:2000—Goat Anti Rabbit HRP Ab (Cat 4040-05, Southern Biotech, USA). Primary GAPDH mouse monoclonal Ab at 1:1000 (SC-365062, Santa Cruz) and Secondary: 1:2000—Goat Anti Mouse HRP (Cat 1010-05, Southern Biotech, USA). SuperSignal West Pico PLUS substrate (Thermofisher, USA) or Clarity Western ECL Substrate (BioRad, USA) was used as the chemiluminescence signal reagent. Images were captured using G: Box Chemi XRQ gel documentation system using GeneSys software version 1.8.3 (Syngene, Cambridge, UK), then Gene Tools software version 4.3.14 was used for analysis of the blots. Full-length blots can be found in supplemental Figure S1.

### Real time-quantitative PCR analysis

Total RNA was extracted using E.Z.N.A. Totally RNA Kit (R6812-01, omega bio-tek, Norcross, GA). cDNA was synthesized using 1 µg of total RNA using a Roche transcriptor first strand cDNA synthesis kit (04896866001, Thermofisher). PowerUp SYBR Green master mix (A25742, Thermofisher) was used as the fluorescent dye and samples were ran in the Cielo 3 RT-qPCR machine (Azure Biosystems, Dublin, CA). Absolute qPCR was done using standard serial dilutions of cDNA to create a standard curve. Primers were designed using NCBI Primer-BLAST and were purchased from IDT Technologies (Coralville, IA).

The following human primer sequences were used:

**Table:** 

Gene	Forward (5′–3′)	Reverse (5′–3′)
GAPDH	AGGTCGGTGTGAACGGATTTG	TGTAGACCATGTAGTTGAGGTCA
hCG-β	TCTGTGCCGGCTACTGCCCC	TTGGGACCCCCGCAGTCAGT

### ELISA assay

Cultured media of each cell line was collected after 72 h posttreatment using 4 µM concentrations of control and LK-1 and stored at −20 °C until assayed. Human hCG-beta ELISA Kit (EH23RB, Thermofisher, USA) was used to determine hCG-β knockdown and expression in each cell line according to the manufacturers’ instructions. Absorbance at 450 nm was measured using BMG Labtech FLUOstar OPTIMA microplate reader (Cary, North Carolina, USA).

### Live cell fluorescence microscopy

Cells were plated in a 24-well plate at 5 × 10^4^ cells/well in complete media and allowed 24 h to adhere to the plate prior to treatment. After 24 h, the media was replaced with reduced serum of 0% FBS media and 4 µM of control or LK-1 treatment was added. The cells were allowed to incubate for 72 h before images were taken. CellMask™ Plasma Membrane Stain (green) (ThermoFisher, USA) was used to dye the cell membranes according to manufacturer’s instructions. Propidium Iodide ReadyProbes™ Reagent was used to stain dead cells according to manufacturer instructions (ThermoFisher, USA). After the stains were added and mixed in the media, they were incubated for 20 min before they were washed with PBS twice before adding Live Cell Imaging Solution media (A59688DJ, ThermoFisher, USA). The cells were imaged using a Leica DMIL LED microscope and Thor Labs monochrome camera attached to the microscope. The images were then analyzed using ImageJ software.

### 2D colony formation assay

Cells were seeded in 6-well, surface-treated culture plates at a density of 2000 cells per well, except for HCC1937 cells, which were seeded at 4000 cells per well. Cells were initially cultured in medium containing 10% fetal bovine serum (FBS). Following an overnight incubation, the media was replaced with 5% FBS medium for all cell lines, except for JEG-3 and HCC1395, which remained in 10% FBS. Cells were incubated for an additional 24 h before treatment. For treatment, 4 µM of either control or LK-1 PMO was added to each well, and cells were incubated with treatments for one week. PMOs were removed and cells were maintained in culture for up to three weeks to monitor post-treatment effects. For staining, cells were fixed with 0.2% methylene blue and incubated overnight at 4 °C. Plates were subsequently rinsed with water and air-dried. Images of the stained plates were captured using the G Chemi XRQ gel documentation system (Syngene, Cambridge, UK) with GeneSys software version 1.8.3.

### Wound healing/scratch invasion assay

Cells were seeded onto a 6-well, surface-treated tissue culture plate at a density of 2.5 × 10^5^ cells per well in media containing 10% fetal bovine serum (FBS) and cultured to maximum confluency. A scratch was then created in each well by drawing a 200 µL pipette tip in a straight line across the confluent cell layer to generate a stripe of unoccupied space. Following the scratch, 4 µM of either control or LK-1 treatment was applied in media containing 1% FBS, except for MCF-7 cells, which were treated in 5% FBS media. Treatments were applied for only 24 h, after which the wells were washed with phosphate-buffered saline (PBS) to remove treatments and non-adherent dead cells. Brightfield images of the scratch area were then captured at 5× magnification using a Leica DMIL LED microscope equipped with a ThorLabs camera. The plate was subsequently incubated, and every 24 h, a PBS wash was performed followed by the addition of fresh low-FBS media. Brightfield images were taken after each media change. The images were processed using ImageJ software to manually segment the scratch area, enabling calculation of the cell migration area.

### 3D tumor spheroid assay

Cells were seeded in a 6-well plate at a density of 2.0 × 10^5^ cells per well in medium containing 10% fetal bovine serum (FBS) and incubated overnight. The following day, 20 µL of Nanoshuttle-PL (Greiner Bio-One, VWR, USA) were added to each well, and cells were incubated overnight to facilitate magnetic labeling.

After incubation, the media was aspirated, and cells were washed with 1 mL of phosphate-buffered saline (PBS) to remove excess Nanoshuttle. Cells were harvested using trypsin, counted, and seeded at a density of 4000 cells per well into a 96-well cell-repellent plate. A magnetic drive was positioned under the plate to aggregate cells into spheroids. Cells were incubated for 1 h, then imaged at 10X magnification using a brightfield Leica DMIL LED microscope to confirm consistent seeding. The plate was then returned to the incubator for 24 h.

Spheroid formation was visually confirmed the following day. At this stage, treatments were prepared by adding 4 µM of either control PMO or LK-1 in medium without FBS. Both treatments were modified with a FITC fluorescein tag on the 5’ end for tracking. Propidium Iodide (PI) ReadyProbes™ Reagent (Invitrogen, Thermo Fisher Scientific, USA) was also added (1 drop per well) to each treatment condition, and a 20-min incubation was allowed for PI intercalation before imaging. Fluorescent and brightfield images of each spheroid were captured at 10X magnification using fluorescent microscopy. After imaging, the plate was returned to the incubator for 24 h. Subsequently, 100 µL of medium was removed from each well and replaced with fresh 0% FBS medium to dilute background fluorescence from PI and FITC. Spheroids were then re-imaged using both fluorescent and brightfield microscopy, repeating imaging every 24 h. Fresh 4 µM treatments were reintroduced into each respective well at each imaging point. Imaging continued every 24 h to monitor spheroid responses, until the completion of the experiment.

### Cell cycle analysis

SCaBER or HCC1395 cells we treated with or without LK-1 or control PMO as described above. Cells were harvested and incubated in 1 ml complete media containing 2 µl Vybrant DyeCycle Violet (Thermo Fisher) at 37℃ for 30 min. Cells were then analyzed by flowcytometry using a BD Canto II flow cytometer equipped with 3 lasers. Live cells were determined by forward scatter and side scatter analysis. Fluorescence intensity of the Vybrant DyeCycle Violet on single cells was viewed on a linear scale. Histogram plots were generated using FlowJo.

### Data analysis

Data was analyzed using Prism software (Version 8.1, Graphpad Software, La Jolla, CA) and specific statistical analysis is described in the each figure legend. P values less than 0.05 were considered statistically significant.

## Supplementary Information

Below is the link to the electronic supplementary material.

Supplementary Material 1

## Data Availability

The datasets generated during and/or analyzed during the current study are available from the corresponding author on reasonable request.
