# Supplementary figures and images for: LK-1: an investigational therapy targeting hCG-β in metastatic breast, bladder, ovarian, and cervical cancers
Source: Sci Rep. 2026 Feb 20;16:10061. doi: 10.1038/s41598-026-38909-6 (PMC13021918; doi:10.1038/s41598-026-38909-6)

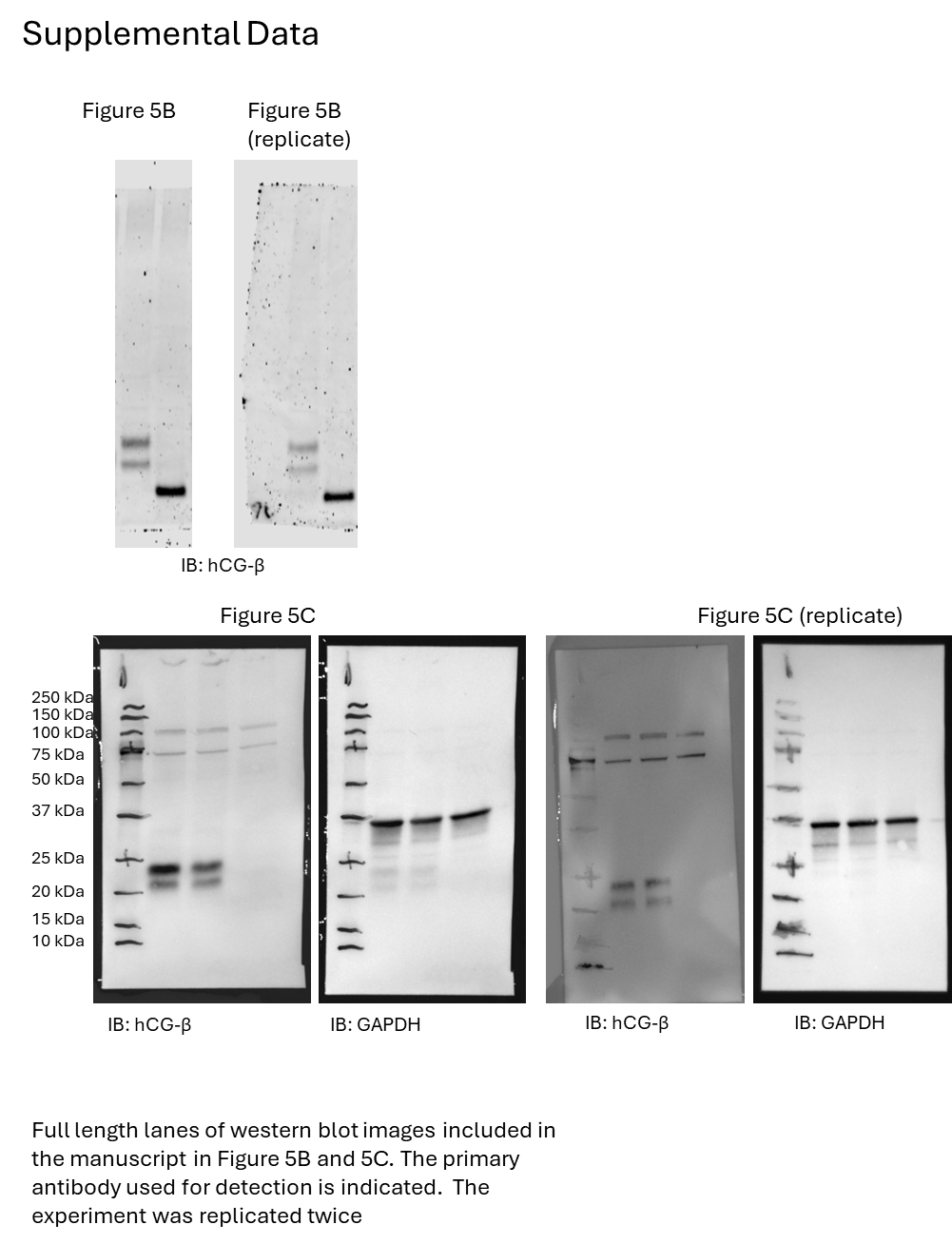

Supplement: Supplementary file 1 — Supplementary Material 1 [file 41598_2026_38909_MOESM1_ESM.docx]
